# Host genotype affects endotoxin release in excreta of broilers at slaughter age

**DOI:** 10.3389/fgene.2023.1202135

**Published:** 2023-06-08

**Authors:** F. Marcato, J. M. J. Rebel, S. K. Kar, I. M. Wouters, D. Schokker, A. Bossers, F. Harders, J. W. van Riel, M. Wolthuis-Fillerup, I. C. de Jong

**Affiliations:** ^1^ Wageningen Livestock Research, Wageningen University and Research, Wageningen, Netherlands; ^2^ Wageningen Bioveterinary Research, Lelystad, Netherlands; ^3^ Institute for Risk Assessment Sciences, Utrecht University, Utrecht, Netherlands

**Keywords:** broiler, genetic strain, probiotics, early feeding, microbiome, endotoxin, performance

## Abstract

Host genotype, early post-hatch feeding, and pre- and probiotics are factors known to modulate the gut microbiome. However, there is a knowledge gap on the effect of both chicken genotype and these dietary strategies and their interplay on fecal microbiome composition and diversity, which, in turn, can affect the release of endotoxins in the excreta of broilers. Endotoxins are a major concern as they can be harmful to both animal and human health. The main goal of the current study was to investigate whether it was possible to modulate the fecal microbiome, thereby reducing endotoxin concentrations in the excreta of broiler chickens. An experiment was carried out with a 2 × 2 × 2 factorial arrangement including the following three factors: 1) genetic strain (fast-growing Ross 308 vs. slower growing Hubbard JA757); 2) no vs. combined use of probiotics and prebiotics in the diet and drinking water; and 3) early feeding at the hatchery vs. non-early feeding. A total of 624 Ross 308 and 624 Hubbard JA757 day-old male broiler chickens were included until d 37 and d 51 of age, respectively. Broilers (*N* = 26 chicks/pen) were housed in a total of 48 pens, and there were six replicate pens/treatment groups. Pooled cloacal swabs (*N* = 10 chickens/pen) for microbiome and endotoxin analyses were collected at a target body weight (BW) of 200 g, 1 kg, and 2.5 kg. Endotoxin concentration significantly increased with age (*p* = 0.01). At a target BW of 2.5 kg, Ross 308 chickens produced a considerably higher amount of endotoxins (*Δ* = 552.5 EU/mL) than the Hubbard JA757 chickens (*p* < 0.01). A significant difference in the Shannon index was observed for the interaction between the use of prebiotics and probiotics, and host genotype (*p* = 0.02), where Ross 308 chickens with pre-/probiotics had lower diversity than Hubbard JA757 chickens with pre-/probiotics. Early feeding did not affect both the fecal microbiome and endotoxin release. Overall, the results suggest that the chicken genetic strain may be an important factor to take into account regarding fecal endotoxin release, although this needs to be further investigated under commercial conditions.

## 1 Introduction

In recent years, the Netherlands has shifted toward a new broiler production system. This system uses slow-growing broiler strains housed at reduced stocking densities (25–38 kg/m^2^) compared to the conventional production system using fast-growing broiler strains with a stocking density of 38 kg/m^2^ or higher (Bracke et al., 2019; Vissers et al., 2021; de Jong et al., 2022). This change may have an impact on the amount of endotoxins released in the excreta of these animals, which is a societal concern. Endotoxins, also referred to as lipid A in lipopolysaccharides, are a major component of the outer membrane of Gram-negative bacteria (Reisinger et al., 2020). The amount of endotoxins released in the environment relies on the balance between different microbial communities (especially between Gram-positive and Gram-negative bacteria) in the gastrointestinal tract (GIT) of broiler chickens (Hou et al., 2016; Yadav and Jha, 2019; Zhu et al., 2019; Reisinger et al., 2020). As a result of both infectious and non-infectious stressors, the balance of the gut microbiome can be disrupted (overgrowth of particular bacterial species, including γ-proteobacteria bloom), leading to changes in the GIT, such as a reduction in intestinal barrier function, poor nutrient digestibility, and, thus, an increased risk of bacterial translocation and endotoxin release in the excreta (Lapa e Silva et al., 2000; Shang et al., 2018). A high release of endotoxins in the droppings of broilers and a possible increase in *E. coli* can trigger local inflammatory processes in the respiratory tract that can compromise lung health, leading to chronic bronchitis, non-allergic asthma, and pneumonia in both humans and animals (Lapa e Silva et al., 2000; Basinas et al., 2012). The major concern is the release of endotoxins in the excreta, and consequently, in the litter, which can end up in the air with fine dust, representing a public health concern for the farmers and residents surrounding poultry farms (Basinas et al., 2012; Hagenaars et al., 2017). Moreover, in commercial broiler production, performance and health are important and their impairment can also lead to a severe economic impact on the broiler industry (Schokker et al., 2021). As reported in the literature, the genotype of the host (including the difference between fast-growing strains and slow-growing strains) can have a direct effect on microbiota composition, for example, through secretions into the gut, control of gut motility, and modification of epithelial cell surfaces (Zhao et al., 2013; Schokker et al., 2015). These changes in the microbiota composition can influence intestinal morphological and immunological development, feed conversion, nutrient absorption, and manure quality. However, in broiler production, there is a knowledge gap on the effect of the genetic strain on the release of endotoxins in the excreta. Moreover, there is a need to find potential solutions, which can modulate the gut microbiome by steering the bacterial niche to attain a lower Gram-negative bacteria load in the intestine and a lower release of endotoxins in the excreta.

Recent studies have shown that different factors can affect the gut microbiome composition (Schokker et al., 2021), which, in turn, can affect endotoxin release in the excreta. These factors include age (Ballou et al., 2016), sex (Lee et al., 2017), feed (Oakley et al., 2014; Reisinger et al., 2020; Perricone et al., 2023), management, i.e., housing or type of bedding (Wang et al., 2016), the environment, i.e., hygiene level in the farm, and genetic background (Siegerstetter et al., 2017). Nutritional interventions are known to have effects on the microbiome, and health and performance of broilers chickens (Froebel et al., 2020). Two important nutritional strategies to affect gut health and performance are the addition of probiotics and/or prebiotics to commercial diets (Jha and Berrocoso, 2015; Yadav and Jha, 2019) and the provision of early feeding at the hatchery (Jha et al., 2019). Probiotics are defined as live non-pathogenic microorganisms that can confer a health benefit on the host (Ritzi et al., 2014), whereas prebiotics are non-digestible fibrous nutrients that stimulate the growth of (beneficial) bacteria within the intestinal microbiota (Taha-Abdelaziz et al., 2018). Administration of probiotics in the first week of life of chickens can affect the microbiome composition of broilers in the long term by increasing the diversity and abundance of *Lactobacillus* (Nakphaichit et al., 2011). Moreover, a combination of probiotics and prebiotics in poultry diets has been shown to improve growth performance, feed efficiency, gut development, and microbial balance in chickens (Haghighi et al., 2005; Brisbin et al., 2008; Zhang and Kim, 2014). Provision of feed immediately post-hatch (early feeding) promotes early gut development (Reicher et al., 2020) and provides dietary antigens, which influence microbial diversity and colonization pattern in the chicken gut (Simon et al., 2015). Moreover, early feeding can also positively affect the performance of broiler chickens in the long term until slaughter age (Hollemans, 2020).

Despite the importance of these factors, effects of genetic strain, combined with the use of prebiotics and probiotics, and early feeding, and especially their interactions on the interplay between the fecal microbiome, release of endotoxin, and health and performance of broilers, have hardly been investigated. Therefore, the main aim of the current study is to investigate the impact of three different factors, i.e., genetic strain, combined use of probiotics and prebiotics and early feeding, and their possible interaction with the fecal microbiota and concentration of endotoxins in broiler excreta. In addition, the effects of these factors on the performance [body weights, daily body weight gain (BWG), feed conversion ratio (FCR), and average daily feed intake (ADFI)] and welfare of broilers [footpad dermatitis (FPD), hock burn (HB), cleanliness, and gait score] are also investigated to determine whether the intervention had any positive or negative effect on these. Our hypothesis is that single factors, especially the use of prebiotics and probiotics or early feeding, have an effect on the microbiome. The genetic strain is also expected to have an impact. Moreover, the combination of some of these factors and, in particular, the use of slow-growing chickens in combination with the use of pre-/probiotics and early feeding is expected to affect the microbiome by reducing the abundance of Gram-negative bacteria in the gut, thus creating a shift in the ${Gram}^{-}/{Gram}^{+}$ ratio and thereby contributing to a reduction in the amount of endotoxins released in the excreta.

## 2 Materials and methods

### 2.1 Experimental design

The experiment was set up as a complete 2 × 2 × 2 factorial arrangement with the following three factors: 1) chicken genetic strain (fast-growing Ross 308 vs. slow-growing Hubbard JA757); 2) no use vs. combined use of probiotics and prebiotics in the diet and the drinking water; and 3) early feeding at the hatchery vs. non-early feeding. Therefore, the combination of these treatments resulted in eight treatment groups:

R + Pro + EF = Ross 308 + probiotics + early feeding.

R + Pro − EF = Ross 308 + probiotics + non-early feeding.

R − Pro + EF = Ross 308 − probiotics + early feeding.

R − Pro − EF = Ross 308 − probiotics + non-early feeding.

H + Pro + EF = Hubbard JA757+ probiotics + early feeding.

H + Pro − EF = Hubbard JA757 + probiotics + non-early feeding.

H − Pro + EF = Hubbard JA757 − probiotics + early feeding.

H − Pro − EF = Hubbard JA757 − probiotics + non-early feeding.

The study complied with the Dutch law on animal experiments. The project was approved by the Central Commission on Animal Experiments (license number AVD4010020197985; experiment no. 2019. D-0009.002), and the experiment was approved by the Ethical Committee of Wageningen University and Research, Netherlands. The experiment was conducted at the experimental research facility of Wageningen University and Research.

### 2.2 Allocation of broilers to the treatment groups and housing management

A total of 624 fast-growing (Ross 308; breeder age of 45 weeks) and 624 slow-growing (Hubbard JA757; breeder age of 45 weeks) day-old male broiler chickens were obtained from a commercial hatchery (Probroed and Sloot, Lunteren, Netherlands). Prior to hatching, on day 18 of incubation, the eggs were already divided into eight treatment groups (*N* = 156 eggs per group), as indicated previously. Contact between chicks belonging to different treatment groups was avoided from this moment until the end of the experiment in order to prevent contamination. The chicks were transported in 16 crates (*N*= 78 chicks/crate; two crates per treatment group) from the hatchery to the experimental facility, and the duration of the journey was approximately 1 h. Upon arrival at the research facility, the chicks were neck-tagged for individual identification and randomly allocated to their respective pen. Two identical climate-controlled rooms, each containing 24 pens, were used to house the chickens. The broilers were allocated to 48 pens (26 chickens/pen) according to a completely randomized block design, which consisted of six blocks of eight pens equally distributed in the two rooms. Each pen measured 1.10 × 1.90 m (L × W) and was provided with wood shavings (2.0 kg/m^2^) and one metal perch (length: 150 cm; height: 2 cm). The current experiment used only one stocking density, which was lower (26–28 kg in the last phase of the experiment) than the stocking density used in the current industry due to the utilization of small pens. Feed and water were provided *ad libitum* for all treatment groups throughout the whole experiment. Feed was provided via a round feeder (diameter: 35 cm) hanging in the pen. Water was provided via seven nipples along the side wall of the pen. On the day of arrival of the chicks at the research facility, the temperature was on average 33.3°C in the rooms, and it gradually declined to 20°C on day 51. A continuous light program was applied during the first 3 days and was thereafter changed to 18L:6D until the end of the experiment, with a light intensity of 20 lux at bird height. On day 17, chickens were vaccinated against Newcastle disease and on day 22 against infectious bronchitis.

### 2.3 Feeding program and treatments

A three-phase feeding program was applied, and all treatment groups received an identical diet formulated by ForFarmers (Lochem, Netherlands) and produced by Research Diet Services B.V. (Wijk bij Duurstede, Netherlands). The diet was formulated in such a way that it was intermediate to the guidelines for both breeds. A starter diet was provided between days 0 and 14 (ME = 2,934 kcal/kg; CP = 218.2 g/kg; dLys = 12.4 g/kg), a grower diet between days 14 and 37 (ME = 3,023 kcal/kg; CP = 190.3 g/kg; dLys = 10.1 g/kg), and a finisher diet (only provided to Hubbard JA757) between days 37 and 51 (ME = 3,075 kcal/kg; CP = 184.5 g/kg; dLys = 9.6 g/kg). The composition of the diets is given in Supplementary Table S1. Chickens belonging to treatment groups R + Pro + EF, R − Pro + EF, H + Pro + EF, and H − Pro + EF also received a prestarter diet at the hatchery (ME = 3,048 kcal/kg; CP = 211.9 g/kg; dLys = 12.4 g/kg). In addition, all diets for treatment groups R + Pro + EF, R + Pro − EF, H + Pro + EF, and H + Pro − EF included a mixture of prebiotics and probiotics (Biomin GmbH. Getzersdorf, Austria). PoultryStar Hatchery^EU^ was added to the prestarter diet (containing 1 × 10^13^ colony-forming units (CFU)/kg), whereas PoultryStar ME^EU^ (containing 2 × 10^8^ CFU/g) was added to the starter, grower, and finisher diets. To ensure the modulation of the gut microbiome, the mixture of pre/probiotics was also included in the water (PoultryStar Sol^EU^; containing 5 × 10^9^ CFU/g) between days 0 and 3, and around the first diet switch (on days 13 and 14). The prebiotics used in the diet and water were fructooligosaccharides (FOS). All these products contained a mixture of *Bifidobacterium*, *Lactobacillus,* and *Enterococcus*.

### 2.4 Sampling moments and measurements

#### 2.4.1 Fecal microbiome

**FIGURE 1:**
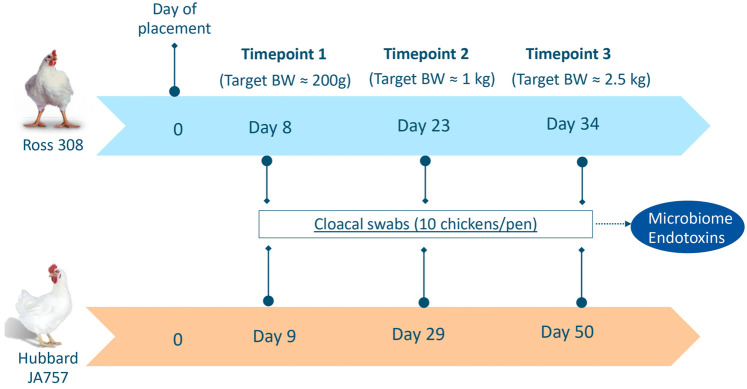
Overview of sampling moments for microbiome and endotoxin analyses in Ross 308 chickens and Hubbard JA757 chickens, respectively. BW = body weight.

Cloacal swabs (pharma regular applicator flocked-tipped in dry tubes, Copan Diagnostics, Brescia, Italy) were collected for both Ross 308 and Hubbard JA757 chickens at three ages based on similar target body weights (BW). Although the two genetic strains were fed the same diet, they had a different growth profile, and thus, it was important to sample them on the same target BW, which resulted in a different age per breed at each sampling moment. The sampling occurred on target BW of around 200 g, 1 kg, and 2.5 kg, which corresponded to days 8, 23, and 34 of age for Ross 308 chickens, and days 9, 29, and 50 of age for Hubbard JA757 chickens, respectively (Figure 1). Swabs were collected from the same 10 broilers randomly selected per pen at all ages, then placed temporarily on dry ice, and stored at −80°C until further analyses. In the case of a dropout, another chicken was selected randomly within the same pen as a replacement. The analyses were carried out at Wageningen Bioveterinary Research where 10 individual samples/pen were pooled together in 2 mL pyrogen-free water with 0.05% Tween-20. Then, 1 mL was used for endotoxin measurement, whereas 1 mL was used to extract microbial DNA from the pellet after centrifugation for 20 min at 20,000 × *g*.

Microbial DNA was isolated using the PureLink Microbiome DNA Purification Kit (Thermo Fisher Scientific) using bacterial pellets suspended in 800 μl lysis buffer. The ballistic DNA extraction method with beads was utilized without lysozyme pretreatment. Following extraction, the DNA extracts were quantified using the Invitrogen™ Qubit™ 3.0 Fluorometer and stored at −20°C for further processing. The hypervariable regions V3+V4 of the 16S rRNA gene were amplified in a limited-cycle PCR in triplicate with the primers CVI_V3-forw 5′CCT​ACG​GGA​GGC​AGC​AG-3′ and CVI_V4-rev 5′-GGACTACHVGGGTWTCT-3′; the following amplification conditions were used as previously described (Jurburg et al., 2019): 98°C for 2 m, followed by 20 cycles of 98°C for 10 s, 55°C for 30 s, 72°C for 10 s, and finally by 72°C for 7 min. The triplicate PCR products were pooled, checked on TapeStation (Agilent, United States), and after barcoding per sample, subsequently sequenced on a MiSeq sequencer (Illumina Inc., San Diego, CA) using a V3 paired-end 300 bp kit.

Sequence processing and statistical analyses were performed in R 3.6.1. (R Core Team, 2020). The amplicon sequences were demultiplexed per sample and subsequently filtered, trimmed, error-corrected, dereplicated, chimera-checked, and merged using the DaDa2 package (v.1.18.0) (Callahan et al., 2016). By using the standard parameters except for *TruncLength* = (270,220), *trimLeft* = (25,33), and *minOverlap* = 10, reads were classified against the SILVA v.138.1 database (Quast et al., 2012).

Subsequent analyses were performed using R 4.0.2. For this experiment, prior to analyses, samples were filtered on *sample_sums* per sample to be equal or higher than 10,000, resulting in one sample being excluded from the data. Thereafter, only for alpha diversity-based analysis, the data were rarefied to 18,796 per sample (*rarefy_even_depth*) with set.seed (12,345). All the other analyses were performed with the full dataset at the ASV level, unless indicated otherwise. The final dataset contained 2,585 amplicon sequence variants (ASVs).

The alpha diversity index was used to estimate the microbiome diversity within microbial communities, and this analysis included Shannon’s diversity index, observed richness, and Pielou’s evenness. Principal coordinate analyses were carried out with the *vegan* package after transformation in a Bray–Curtis dissimilarity matrix. Composition of the community was generated at phylum and genus levels (Supplementary Figures S1, S2).

#### 2.4.2 Endotoxin concentration

An aliquot from pyrogen-free water and Tween was used to elute cloacal swabs (*N* = 144) collected for the analysis on the fecal microbiome, and a small volume (1 mL) was also used for the analysis of endotoxins. Samples were thawed at room temperature and then transferred to 15-mL polypropylene tubes (Greiner Bio-One B.V., Etten Leur, Netherlands) and agitated for 1 h at an end-over-end roller at room temperature. The samples were then centrifuged for 15 min at 1,000 × *g,* and the supernatant was aliquoted and stored frozen at −20°C. Analyses were conducted in glass tubes, rendered pyrogen-free by heating for 4 h at 200°C. Endotoxin content in the samples was analyzed by testing the samples in a 1:1,000 dilution with pyrogen-free water (B. Braun medical B.V., Oss, Netherlands) in a kinetic chromogenic Limulus amebocyte lysate (LAL) assay (Lonza, Verviers, Belgium), as described previously and in accordance with recommendations by Spaan et al. (2008). Endotoxin content was expressed as endotoxin units per ml (EU/mL).

#### 2.4.3 Dry matter (DM) content of litter samples

Litter samples were collected at the same ages indicated for the fecal microbiome and endotoxin analyses. The samples were collected on five locations per pen, thoroughly mixed, and subsequently dried to determine the dry matter content. Dry matter was gravimetrically determined by drying at a constant weight at 103°C (ISO, 6,496).

#### 2.4.4 Performance

Body weight (BW) was recorded at the pen level on days 0, 7, 14, 28, 37, and 51 of age. The average daily feed intake (ADFI) was quantified at both pen and individual levels for all feeding phases (starter: 0–14 days; grower: 14–37 days; and finisher: 37–51 days) and was calculated as the difference between the amount of supplied and remaining feed divided over the number of days constituting each feeding phase. Body weight gain (BWG) was also determined at the individual level for all feeding phases, and it was calculated as the difference between BW on the first and last days of each feeding phase. The feed conversion ratio (FCR), expressed as the ratio between the feed supplied and the weight gain, was also calculated for each feeding phase. Mortality (chickens found dead) and culls (chickens euthanized for ethical reasons) were recorded on a daily basis during the whole experiment (0–51 days). Calculations of performance data were corrected for the weight of chickens that died or were culled during the experiment.

#### 2.4.5 Welfare assessment

Assessment of welfare problems was carried out 1 day before slaughter at a target BW of 2.5 kg, thus on day 36 for Ross 308 chickens and on day 51 for Hubbard JA757 chickens. Footpad dermatitis (FPD), hock burn (HB), and cleanliness were assessed on 10 chickens per pen (the same chickens as randomly selected for the sampling of cloacal swabs), whereas the gait score was evaluated on five randomly selected chickens out of these 10 chickens per pen. Footpad dermatitis and hock burn were scored on a scale from 0 (no lesions) to 4 (severe lesions on the foot or hock) (Welfare Quality, 2009). Cleanliness was scored by inspection of the belly on a scale between 0 (clean) and 3 (very dirty) (Welfare Quality, 2009). Evaluation of the gait score was performed according to Kestin et al. (1992) on a scale from 0 (normal, dexterous, and agile) to 5 (incapable of walking). All observations were performed by two trained observers.

### 2.5 Statistical analyses

Microbiota statistical analyses were performed within the R environment (version 4.1.0), where the following packages were used to calculate alpha and beta diversities: *phyloseq* [version 1.36.0 (McMurdie and Holmes, 2013)], *vegan* [version 2.5–7 (Oksanen et al., 2019)], and *microbiome* [version 1.14.0 (Lahti et al., 2017)]. With regard to alpha diversity, the command *estimate_richness* was used, and the observed richness and Shannon index were selected. To test for differences between the treatment groups, we made pairwise comparisons using a Wilcoxon test. For visualizing the beta diversity, a principal coordinate analysis (PCoA) with Bray–Curtis dissimilarity was first performed. Second, a permutation test for homogeneity of multivariate dispersions (*betadisper*), followed by a permutational multivariate analysis of variance using distance matrices was performed (*adonis*). These were performed for each day separately by testing for the main effects of breed, probiotics, and early feed, and their interactions. Moreover, to evaluate the impact of the experimental factors on the modulation of the intestinal Gram-negative population, bacteria were classified according to their predicted Gram stain based on their taxonomy. Typing the bacteria was performed manually in sequential steps. First, PSORTdb was accessed (16 November 2020) and the file “Experimental-PSORTdb-v2.00-v3.00” was downloaded, which contained 143 organisms with their respective Gram stain. Second, this PSORTdb list was cross-referenced with our bacterial groups (genus level). Third, the portion that was not annotated was manually curated by accessing several online sources other than PSORTdb. A total of 176 bacterial groups were annotated, of which 77 were Gram-negative, 93 Gram-positive, 4 Gram-variable, and 2 could not be determined (Supplementary Table S2). To test for differences between treatment groups on each day, we conducted ANOVA with the main effects of breed, probiotics, and early feed, and their interactions. The input data only contained bacteria that were annotated Gram-negative or Gram-positive, thus excluding the four Gram-variable and two unannotated Gram stain bacteria.

Endotoxin concentration and performance data (BW, BWG, ADFI, and FCR) were continuous response variables and were analyzed with a linear mixed model (LMM). Components of variance were estimated with restricted maximum likelihood (REML), employing procedure MIXED from SAS 9.4 (SAS Inst. Inc., Cary, NC). Residuals were always checked for normality and homogeneity of variance, and variables were log-transformed when required. The linear mixed model for BW (model 1) comprised the following fixed effects in the systematic part of the model (the linear predictor part):

$$\begin{array}{c} µ+Genetic\;{strain}_{i}+{Probiotic}_{j}+Early\;{feeding}_{k}+{Age}_{l}+\left(Genetic\;{strain}_{i}\times {Probiotic}_{j}\right)\\ +\left(Genetic\;{strain}_{i}\times Early\;{feeding}_{k}\right)+\left({Probiotic}_{j}\times Early\;{feeding}_{k}\right)+\left(Genetic\;{strain}_{i}\times {Age}_{l}\right)+\left({Probiotic}_{j}\times {Age}_{l}\right)\\ +\left(Early\;{feeding}_{k}\times {Age}_{l}\right)\;\left(Genetic\;{strain}_{i}\times {Probiotic}_{j}\times Early\;{feeding}_{k}\right)+Ɛ, \end{array}$$ (1)

where µ is the base level, Genetic strain_i_ i = Ross 308 and Hubbard JA757, Probiotic_j_ = mixture of prebiotics and probiotics in the diet (j = yes or no), Early feeding_k_ k = yes or no, and Age_l_ l = days 0, 7, 14, 28, 37, and 51. The model also comprised two- and three-way interactions between the strain, probiotics, early feeding, and age. Interactions were considered not significant when *p* > 0.05 and excluded from the model with the backward elimination procedure. In addition, random effects for the pen and block were included (in the linear predictor). For the pen effects, a first-order auto regressive model (based on the actual distance between different ages) was adopted to introduce a correlation in the model between repeated measurements on the same pen. Here, and in the subsequent analyses, for all fixed effects, approximate F-tests were used (Kenward and Roger, 1997). Interactions that were not significant were excluded from the model (when higher-order interactions were already excluded, i.e., respecting the hierarchy of interaction terms), and subsequent pairwise comparisons were conducted with Fisher’s least significant difference (LSD) method. Endotoxin concentration was analyzed with model 1, but the age effects only included three moments (at a target BW of 200 g, 1 kg, and 2.5 kg, respectively). Variables including BGW, ADFI, and FCR were analyzed per feeding phase (starter: 0–14 days; grower: 14–37 days; and finisher: 37–51 days) with model 2, which comprised the following fixed effects in the systematic part of the model:

$$\begin{array}{c} µ+Genetic\;{strain}_{i}+{Probiotic}_{j}+Early\;{feeding}_{k}+\left(Genetic\;{strain}_{i}\times {Probiotic}_{j}\right)+\left(Genetic\;{strain}_{i}\times Early\;{feeding}_{k}\right)\\ +\left({Probiotic}_{j}\times Early\;{feeding}_{k}\right)+\left(Genetic\;{strain}_{i}\times {Probiotic}_{j}\times Early\;{feeding}_{k}\right)+Ɛ. \end{array}$$ (2)

Both fixed and random effects of model 2 were the same as for model 1 but without the inclusion of age as a fixed factor.

Litter DM content was expressed as a proportion; thus, it was analyzed using a generalized linear mixed model (analysis with penalized quasi likelihood with SAS procedure GLIMMIX), with a logit link function, specifying the “error” variance as a multiple of the binomial variance. Both fixed and random effects of this model were the same as for model 2. Two- and three-way interactions between fixed effects were included in the model, and interactions were considered not statistically significant when *p* > 0.05 and eliminated with the backward procedure. Approximate F-tests (Kenward and Roger, 1997) were used for fixed effects. Subsequent pairwise comparisons were done with Fisher’s LSD method.

Scores of welfare indicators (footpad dermatitis, hock burn, cleanliness, and gait score) were analyzed in R (version 0.1) with a model for ordinal data comprising the same fixed and random effects as model 2.

### 2.6 Data availability

Raw sequencing data have been deposited at NCBI-SRA under bioproject PRJNA975731 and the DaDa2 annotated phyloseq object via Zenodo (https://doi.org/10.5281/zenodo.7965703).

## 3 Results

### 3.1 Effects on the microbiota composition and Gram^−^/Gram^+^ ratio

**FIGURE 2:**
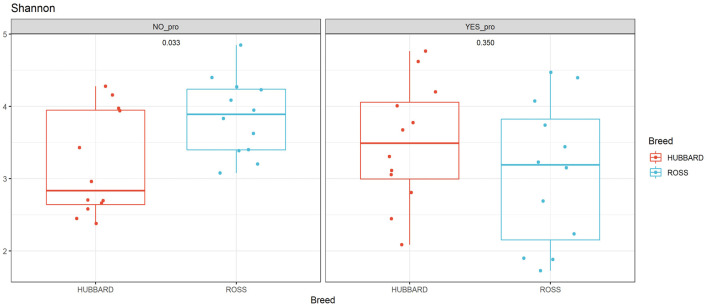
Alpha diversity of fecal microbial samples collected from broiler chickens (fast-growing Ross 308 and slow-growing Hubbard JA757) at a target body weight of 2.5 kg (day 34 for Ross chickens and day 50 for Hubbard chickens, respectively). The figure shows the difference in the Shannon index between the following groups: Ross (NO_pro) = Ross − probiotics; Hubbard (NO_pro) = Hubbard − probiotics; Ross (YES_pro) = Ross + probiotics; and Hubbard (YES_pro) = Hubbard + probiotics.

A trend in the alpha diversity (Shannon index) of the fecal microbiome was observed between Hubbard JA757 and Ross 308 chickens at a target BW of 200 g (day 8 for Ross 308 chickens and day 9 for Hubbard JA757 chickens) (*p* = 0.09; Table 1). Ross 308 chickens tended to have a higher Shannon diversity than Hubbard chickens (Δ = 0.31). At a target BW of 2.5 kg (day 34 for Ross 308 chickens and day 50 for Hubbard JA757 chickens), a trend in the observed species was observed for the interaction between the use of probiotics and early feeding (*p* = 0.09; Table 2) and for the interaction between breed and use of probiotics (*p* = 0.09; Table 2). At the same target BW, a significant difference in the Shannon index was observed for the interaction between the use of probiotics and genetic strain (*p* = 0.02). The group R − Pro had a higher diversity than H − Pro, whereas R + Pro had a lower diversity than H + Pro (Figure 2). Moreover, H − Pro had a lower diversity than H + Pro, whereas R − Pro had a higher diversity than R + Pro (Figure 2). Overall, sampling at a target BW of 2.5 kg resulted in slightly different target BWs between breeds as Ross 308 chickens were less heavy (2.2 kg) and Hubbard JA757 chickens were heavier (2.6 kg), as estimated previously.

**TABLE 1:** Effects of the broiler strain (fast-growing Ross 308 and slow-growing Hubbard JA757), addition of pre/probiotics to the diet, and early feeding on alpha diversity indexes, and relative abundance of Gram-negative bacteria and the Gram^−^/Gram^+^ ratio of the fecal microbiome measured at three different time points.

Parameter	Strain		SD^a^	*p*-value	Probiotics		SD	*p*-value	Early feeding		SD	*p*-value
	Ross 308	Hubbard JA757			Yes	No			Yes	No		
**Time point 1 (target BW^b^ = 200 g)**												
Observed species	439.5	370.0	141.5	0.16	405.2	404.2	141.5	0.98	374.0	435.5	141.5	0.14
Shannon index	3.63	3.32	0.57	0.09	3.47	3.49	0.57	0.92	3.35	3.61	0.57	0.13
^c^Gram-negative (%)	17.0	13.7	13.7	0.42	13.6	17.1	13.7	0.40	15.4	15.3	13.7	0.98
^d^Gram^−^/Gram^+^	0.25	0.18	0.23	0.35	0.18	0.26	0.23	0.31	0.21	0.13	0.23	0.95
**Time point 2 (target BW = 1 kg)**												
Observed species	447.5	441.2	144.7	0.91	450.5	438.2	144.7	0.85	453.7	435.0	144.7	0.66
Shannon index	3.71	3.62	0.58	0.62	3.63	3.71	0.58	0.68	3.68	3.64	0.58	0.79
Gram-negative (%)	17.0	15.4	16.4	0.42	16.5	14.8	16.4	0.40	17.6	14.8	16.4	0.98
Gram^−^/Gram^+^	0.26	0.24	0.31	0.76	0.27	0.23	0.31	0.71	0.30	0.20	0.31	0.60
**Time point 3 (target BW = 2.5 kg)**												
Observed species	424.2	389.7	180.7	0.42	397.7	416.2	180.7	0.66	424.7	389.2	180.7	0.57
Shannon index	3.46	3.34	0.76	0.54	3.28	3.52	0.76	0.27	3.47	3.33	0.76	0.51
Gram-negative (%)	14.1	13.8	12.3	0.42	14.6	13.3	12.3	0.40	16.0	11.9	12.3	0.98
Gram^−^/Gram^+^	0.19	0.19	0.19	0.95	0.20	0.18	0.19	0.60	0.22	0.15	0.19	0.21

^a^ SD= standard deviation;

^b^ BW = body weight.

^c^ Gram-negative (%) = this has been calculated for each sample by taking the sum of all Gram-negative bacteria and Gram-positive and transform these into a percentage.

^d^ Gram−/Gram+ = this has been calculated for each sample by taking the sum of all Gram-negative bacteria and Gram-positive bacteria and subsequently divided Gram-negative by Gram-positive.

**TABLE 2:** Effects of the interaction between the use of pre/probiotics and early feeding, and the interaction between the broiler breeder strain (fast-growing Ross 308 and slow-growing Hubbard JA757) and use of pre/probiotics on alpha diversity indexes of the fecal microbiome measured at slaughter age (target body weight of 2.5 kg).

Parameter	Probiotics × early feeding				SD^a^	*p*-value
	NO × NO	NO × YES	YES × NO	YES × YES		
Observed species	444	388	334	461	180.5	0.09
	Strain × probiotics					
Observed species	Ross 308 × NO	Hubbard JA757 × NO	Ross 308 × YES	Hubbard JA757 × YES	180.5	0.09
	478.0	354.5	370.5	425.0		

^a^ SD= standard deviation.

Beta diversity analysis revealed no significant differences among the treatments and target BWs considered in this study for the tested parameters.

To gain more insights into the microbiota composition per treatment at different time points, stacked bar plots were generated. Regarding the phylum level (Supplementary Figure S1) shows the average relative abundance per individual per day, and regarding genus level the data are presented as average per treatment (Supplementary Figure S2). At the phylum level, there was a clear dominance of *Firmicutes* at all ages (Supplementary Figure S1). At the genus level, the top 10 genera were shown at each age and treatment; however, there were no significant differences in the average relative abundances between treatments (Supplementary Figure S2).

In the current study, the Gram^−^/Gram^+^ ratio and the Gram-negative relative abundance were calculated due to their involvement in the production of endotoxins. However, the results showed no significant differences in both these variables at the different target BWs (Table 1). The results of the PCoA also showed no significant differences between treatments at the different target BWs (Supplementary Figure S3).

### 3.2 Effects on endotoxin release

As a result of the statistical analyses with the model including both age and the interaction age × genetic strain as the main factors, fecal endotoxin release significantly increased with age (*p* < 0.01; Supplementary Table S3), and there was also an interaction age × genetic strain (*p* = 0.01). When observing the analyses per age (thus, without inclusion of age *per se* as the main factor in the model), Hubbard JA757 chickens tended to produce a higher amount of fecal endotoxins (*Δ* = 267 EU/mL) than Ross chickens at a target BW of 200 g (day 8 for Ross 308 chickens and day 9 for Hubbard JA757 chickens) (Table 3). At a target BW of 2.5 kg (day 34 for Ross 308 chickens and day 50 for Hubbard JA757 chickens), Ross 308 chickens produced a considerably higher amount of fecal endotoxins (*Δ* = 552.5 EU/mL) than Hubbard JA757 chickens. Moreover, although not significant, the release of endotoxins in the excreta was lower (*Δ* = −325 EU/mL) when probiotics were fed compared to the diet without their inclusion. Early feeding did not have any significant effect on the release of endotoxins in the excreta (Table 3).

**TABLE 3:** Effects of the broiler strain (fast-growing Ross 308 and slow-growing Hubbard JA757), addition of pre/probiotics to the diet, and early feeding on the endotoxin concentration (EU/mL) in pooled cloacal swabs (N = 144 in total) collected at three time points.

Endotoxins (EU/mL per broiler) measured at	Strain		SEM^a^	*p*-value	Probiotics		SEM	*p*-value	Early feeding		SEM	*p*-value
	Ross 308	Hubbard JA757			Yes	No			Yes	No		
Time point 1 (target BW^b^ = 200 g)	343.6	610.6	99.5	0.06	525.9	428.2	99.5	0.49	410.1	544.1	99.5	0.34
Time point 2 (target BW = 1 kg)	545.9	614.7	143.5	0.64	633.3	527.4	143.5	0.60	634.0	526.6	143.5	0.59
Time point 3 (target BW = 2.5 kg)	1,340.0	787.5	173.9	0.03	901.2	1,226.2	173.9	0.19	1,080.6	1,046.9	173.9	0.89

^a^ SEM= standard error of the mean;

^b^ BW = body weight.

### 3.3 Effects on performance and DM content of the litter

In the current study, mortality was low (2.5% in total); thus, it will not be discussed as it could not be statistically analyzed. Hubbard JA757 chickens had a lower BWG and ADFI, and a higher FCR than Ross 308 chickens during the starter and grower phases (Table 4). The addition of prebiotics and probiotics to the diet contributed to a significantly lower BWG (*Δ* = −6.4 g/d) and ADFI (*Δ* = −11.4 g/d) of Hubbard JA757 chickens in the finisher phase and to a higher ADFI (*Δ* = 0.93 g/d) in the starter phase than the provision of a diet not supplemented (*p* < 0.05; Table 4). The FCR was not affected by the use of probiotics in the diet. Early feeding led to a significantly higher BWG (*Δ* = 2.27 g/d), ADFI (*Δ* = 3.22 g/d), and FCR (*Δ* = 0.03) in the starter phase than non-early feeding (*p* < 0.01; Table 4). A significant three-way interaction age × genetic strain × early feeding was present for BW (*p* = 0.01; Table 5). Moreover, a two-way interaction between breed and early feeding was found for BW. The BW of Ross 308 chickens and early-fed chickens was higher than the BW of Hubbard JA757 and non-early-fed chickens, respectively, at all ages (*p* < 0.01; Table 5). Effects of early feeding on BW were more evident in Ross 308 chickens than in Hubbard JA757 chickens at all ages, whereas the effects of early feeding were only more pronounced at the beginning of the production cycle. A two-way interaction genetic strain × probiotics was present (*p* = 0.03) for ADFI only in the starter phase. Compared with a diet without feed additives, the addition of prebiotics and probiotics to the diet of Ross 308 chickens contributed to a higher ADFI (40.78 vs. 38.98 g/d), whereas there was no difference in ADFI between Hubbard JA757 chickens fed with or without prebiotics and probiotics (31.31 vs. 31.27 g/d). An interaction genetic strain × early feeding was present (*p* < 0.01) for BWG and ADFI only in the starter phase. Early-fed chickens had a higher BWG (35.78 vs. 32.30 g/d for Ross 308 chickens; 25.50 vs. 24.45 g/d for Hubbard JA757 chickens) and ADFI (42.29 vs. 37.47 g/d for Ross 308 chickens; 32.10 vs. 30.47 g/d for Hubbard JA757 chickens) than non-early-fed chickens, and both BWG and ADFI were higher in Ross 308 chickens than in Hubbard JA757 chickens. With regard to the litter, Supplementary Table S4 shows that there was no treatment effect on the DM content of the litter at each target BW.

**TABLE 4:** Effects of the broiler strain (fast-growing Ross 308 and slow-growing Hubbard JA757), addition of pre/probiotics to the diet, and early feeding on the daily body weight gain (BWG), average daily feed intake (ADFI), and feed conversion ratio (FCR) measured at different feeding phases (LS means± SEM^1^).

Parameter	Strain		SEM	*p*-value	Probiotics		SEM	*p*-value	Early feeding		SEM	*p*-value
	Ross 308	Hubbard JA757			Yes	No			Yes	No		
BWG (g/d per broiler)												
Starter (0–14 days)	34.05	24.98	0.28	<0.01	29.80	29.21	0.28	0.14	30.64	28.37	0.28	<0.01
Grower (14–37 days)	76.76	57.67	0.67	<0.01	67.25	67.18	0.67	0.94	68.14	66.29	0.67	0.06
Finisher (37–51 days)	—	71.59	1.02	—	68.42	74.77	1.45	<0.01	71.39	71.79	1.45	0.85
ADFI (g/d per broiler)												
Starter (0–14 days)	39.88	31.29	0.28	<0.01	36.05	35.12	0.28	0.02	37.19	33.97	0.28	<0.01
Grower (14–37 days)	114.4	98.63	1.85	<0.01	106.63	106.39	1.85	0.93	106.74	106.29	1.85	0.86
Finisher (37–51 days)	—	106.80	2.17	—	101.11	112.48	3.07	0.01	108.42	105.18	3.07	0.46
FCR												
Starter (0–14 days)	1.18	1.27	0.008	<0.01	1.24	1.22	0.008	0.26	1.24	1.21	0.008	<0.01
Grower (14–37 days)	1.50	1.72	0.03	<0.01	1.62	1.61	0.03	0.83	1.59	1.63	0.03	0.30
Finisher (37–51 days)	—	1.48	0.03	—	1.47	1.50	0.04	0.67	1.52	1.46	0.04	0.32

^a^ SEM= standard error of the mean.

**TABLE 5:** Effects of time, broiler strain (fast-growing Ross 308 and slow-growing Hubbard JA757), and early feeding, and their interactions on the body weight (BW) of male broilers at different ages (LS means± SEM^1^).

Parameter	Time						SEM	*p*-value
	Day 0	Day 7	Day 14	Day 28	Day 37	Day 51		
**Time**								
BW (g)	44.39	176.41	461.60	1,268.53	2018.80	2,727.12	7.18	<0.01
**Strain × time**							10.15	<0.01
BW Ross 308	46.33	195.69	527.42	1,462.83	2,308.11			
BW Hubbard JA757	42.46	157.12	395.78	1,074.23	1,729.48			
**Early feeding × time**							10.15	<0.01
YES	46.10	186.94	479.56	1,305.55	2,059.89			
NO	42.69	165.87	443.64	1,231.51	1,977.70			
**Strain × early feeding × time**							14.0	0.01
Ross 308 × YES	48.32	211.83	554.41	1,516.22	2,376.31			
Ross 308 × NO	44.33	179.55	500.44	1,409.45	2,239.92			
Hubbard JA757 × YES	43.87	162.05	404.72	1,094.89	1,743.48			
Hubbard JA757 × NO	41.04	152.19	386.84	1,053.57	1,715.48			

^1^ SEM= standard error of the mean.

### 3.4 Effects on welfare indicators

The genetic strain had a significant effect on the cleanliness score at a target BW of 2.5 kg (day 34 for Ross 308 chickens and day 50 for Hubbard JA757 chickens) (*p* < 0.01). The proportion of Ross 308 chickens with cleanliness scores 1 and 2 was higher (52.9% vs. 8.3% and 33.3% vs. 24.1%, respectively) than that of Hubbard JA757 chickens; in contrast, the proportion of Hubbard JA757 chickens with cleanliness score 3 was higher (66.6% vs. 3.3%) than that of Ross 308 chickens; thus, Hubbard JA757 chickens were dirtier than Ross chickens. The cleanliness and footpad dermatitis scores were influenced by the use of prebiotics and probiotics in the diet (both *p* < 0.01). Chickens fed with the diet with prebiotics and probiotics were dirtier and had more footpad dermatitis. The proportion of chickens with cleanliness score 3 was higher for the group receiving a diet with prebiotics and probiotics (39.6% vs. 30.4%) than for the group with a diet without their inclusion. With regard to footpad dermatitis, the proportion of chickens with scores 3 and 4 was higher for the group receiving a diet with prebiotics and probiotics (38.3% vs. 25.4% and 6.7% vs. 0.8%, respectively) than for the group with a diet without their inclusion. Early feeding affected the cleanliness (*p* = 0.01) and gait scores (*p* < 0.01). The proportion of chickens with cleanliness score 3 was higher; thus, chickens were dirtier in the early-fed group (38.3% vs. 31.7%) than those in the group without early feeding. Moreover, the proportion of chickens with gait scores 3 and 4 was higher (i.e., their gait score was worse) for the early-fed group (28.3% vs. 24.6% and 5.4% vs. 2.1%, respectively) than for the group without early feeding.

## 4 Discussion

When analyzing the individual factors, genetic strain was the one with the biggest impact on endotoxin release according to the results of this study, and it will be discussed first. In the current study, the genetic strain affected the release of endotoxins in broiler excreta at a target BW of 2.5 kg (day 34 for Ross 308 chickens and day 50 for Hubbard JA757 chickens), with particularly fast-growing chickens having significantly higher fecal endotoxin levels than slow-growing chickens. To the best of our knowledge, this is one of the first studies to compare endotoxin release between these two host genotypes fed an identical diet under the same experimental conditions. Previous studies focused on the determination of endotoxins in broilers in response to feeding treatments (Gao et al., 2021; Perricone et al., 2023) or heat stress (Reisinger et al., 2020). In addition, one study conducted by Cheng et al. (2004) focused on endotoxin stress responses in two lines of White Leghorn chickens selected for high or low production and survivability, but there are no data available on the comparison between fast-growing and slow-growing broiler chicken genotypes. Our results suggest that endotoxin release in broilers may be influenced by the host genotype. In the present trial, we performed measurements under controlled conditions and with the same diet for both breeds, which is not comparable to commercial practice. For this reason, the results of the current study indicate that further research under commercial conditions is required. Despite the relevance of these findings, more research is needed to quantify the production of endotoxins in both Hubbard JA757 and Ross 308 chickens in a commercial setup under the current practices. The study was conducted on a relatively small scale and under experimental conditions, where both breeds received an identical diet not meeting the full growth potential of the broilers; thus, effects obtained in a commercial setting might be different. The broiler genetic strain has also been recognized as a factor with potential influence on intestinal microbiota composition (Schokker et al., 2015; Han et al., 2016; Kers et al., 2018; Ji et al., 2020), which, in turn, might affect endotoxin release. Despite the difference in endotoxin release between the two breeds at a target BW of 2.5 kg, this study showed no difference in the observed species, Shannon index, Gram^−^/Gram^+^ ratio, and the Gram-negative relative abundance between Ross 308 and Hubbard JA757 chickens. Although we performed fecal microbiota analysis, our finding is in line with previous reports, where researchers observed no significant difference in fecal microbiota composition among Ross and Hubbard chickens (Richards et al., 2019; Tůmová et al., 2021). In other studies where the microbiota composition in different genetic strains of chickens was studied (Zhao et al., 2013; Meng et al., 2014; Mignon-Grasteau et al., 2015; Kers et al., 2018), Hubbard chickens were hardly included in these comparisons. Despite the lack of significant effects on the microbiome, there was still a difference in the endotoxin release between the two broiler strains. This can be explained by a higher amount of *Ruminococcus* and *Lachnoclostridium* bacteria found in Hubbard JA757 chickens than in Ross 308 chickens at a target BW of 2.5 kg, which are primarily butyrate producers involved in the maintenance of intestinal cell integrity (Timbermont et al., 2010; Antonissen et al., 2016).

The current study suggests that genetic strain in combination with feeding prebiotics and probiotics plays an important role in the modulation of the microbiome composition in the feces of chickens at a target BW of 2.5 kg, with a favorable effect only on Hubbard JA757 chickens. Addition of these feed additives to the diet and drinking water contributed to an increased fecal microbial diversity in Hubbard JA757 chickens than in Ross 308 chickens. However, it should be noted that these results might have been influenced by age differences (34 vs. 50 days) and actual BW differences (2.2 vs. 2.6 kg) between the two strains of chickens. As reported by previous studies (Lee et al., 2017; Feye et al., 2020; Bindari and Gerber, 2022), the GIT microbial composition changes with age, where both BW and microbial richness are entangled, which, in turn, affects endotoxin release. It is thus necessary to conduct more research in the future to further investigate the link between endotoxin production and microbial composition in both Ross 308 and Hubbard JA757 chickens. An ideal scenario would be to include all comparisons of similar ages and BW for a more extensive interpretation of the microbial/endotoxin data. Moreover, our study suggested an association between endotoxins and fecal community composition; this association was not analyzed yet, but it could be tested in future studies to better understand the mechanisms linking changes in the gut microbiome composition and endotoxin release.

Contrary to our expectations, the feeding interventions did not result in changes in endotoxin release and differences in fecal microbiota. The immediate provision of feed after hatching (early feeding) is thought to have many beneficial effects, including enhancing the intestinal functionalities/gut health and stimulating intestinal microbial colonization in early life (Simon et al., 2015; Hollemans et al., 2018). As reported by Rubio (2019), the establishment of an adequate intestinal microbiota is essential for the production of antibodies and for the stimulation of early maturation of the cellular components of the intestinal immune system. However, in this study, both fecal microbiome and endotoxin release were not affected by early feeding. Whether intestinal immune development was affected was not investigated.

The mixture of prebiotics and probiotics contributed to a higher ADFI in the starter phase, and the interaction between the use of these feed additives and genetic strain showed that the effect was only present in Ross 308 chickens. These results are in line with previous studies (Awad et al., 2009; Flores et al., 2016; Askelson et al., 2018; Froebel et al., 2020). Despite the beneficial effects of prebiotics and probiotics in the starter phase, these feed supplements had a negative effect on BWG and ADFI of Hubbard JA757 chickens in the finisher phase. These results are difficult to explain because to the best of our knowledge, there are no studies conducted in Hubbard chickens and, thus, supporting the current findings. Perhaps the increase in fecal microbial richness, as shown in our study, of Hubbard JA757 around slaughter weight in combination with the use of probiotics has created a larger competition for nutrients, which might have offset some of the beneficial effects of probiotics on nutrient digestibility and absorption, leading to a worse performance (Olnood et al., 2015). Addition of prebiotics and probiotics also contributed to more severe scores of footpad dermatitis and to dirtier feathers at the end of the production cycle. Footpad lesions can be triggered by nutritional imbalances and more frequently by litter moisture (Mayne, 2005). Addition of an extra drinking bowl in the middle of the pen on these days might have contributed to more spillage of water, thus affecting the litter quality. Another explanation for the differences in footpad lesions might be related to the longer time spent by Hubbard JA757 chickens in their pens. These chickens were slaughtered at an older age; thus, their feet and plumage might have been in contact with a litter of worse quality for a longer time as well, causing worse welfare scores.

The effects of early feeding on the long-term performance of broilers are controversial. Some studies showed a beneficial effect (de Jong et al., 2017; da Silva et al., 2021), whereas other studies reported no effect in the long-term (Simon et al., 2014; Hollemans et al., 2018). In line with previous research (de Jong et al., 2017; da Silva et al., 2021), the current study showed that early feeding did positively affect the BWG, ADFI, and FCR in the starter phase. These results underline the importance of the provision of feed to broiler chicks immediately at the hatchery as the delay in feeding time can have negative consequences on the performance of these chickens in the early stages of production. In line with previous studies (de Jong et al., 2017; Hollemans, 2020), BW of early-fed chickens in the current study was always higher than the BW of non-early-fed chickens until day 37 of age. In general, the variation in the BW between early-fed and delayed-fed chicks depends on the experimental context and feed deprivation times, and the results of the current study seemed to confirm that early feeding has long-term effects on the absolute values of BW (Hollemans, 2020). However, the current study also showed that early feeding resulted in worse cleanliness and gait scores at the slaughter age compared to delayed nutrition. This could be a consequence of the relatively small pens in combination with the heavier weight of the early-fed broilers than the non-early-fed broilers. However, this requires further investigation as Giersberg et al. (2021) did not find any differences in both plumage and gait scores between early-fed and non-early-fed Ross chickens. Another study on Ross chickens (Lingens et al., 2021) showed that early feeding contributed to a higher BW of broilers throughout the rearing period and to significantly lower FPD scores between days 14 and 32 than in not-early-fed broilers. Different studies (Wijnen et al., 2021; Wijnen, 2022) also suggest that early-fed broilers have a higher resilience (in terms of higher disease resistance and tolerance, as well as higher capability to recover from diseases) than delayed-fed broilers, which was not investigated in this study. Overall, early nutrition can be advantageous in terms of the performance of the broilers, but, as shown by the results of the current study, it might also lead to more welfare problems linked to a higher body weight at a later production stage (although these results were based on experimental conditions). Due to the controversial results found in the literature, it is thus not clear yet whether this practice has long-term beneficial effects for broiler chickens, and it might be interesting to investigate whether early nutrition can affect other aspects, for example, behavior or immunity, in order to be able to draw stronger conclusions on this practice.

## 5 Conclusion

The current study was the first to demonstrate the effects of host genetics and feed interventions and, in particular, their interaction, on endotoxin release, the fecal microbiome, and on broiler performance and welfare. The current study showed that, by using a different host genotype, significantly lower concentrations of endotoxins can be achieved at a target BW of 2.5 kg under controlled conditions with the same diet for both breeds. Early feeding in the hatchery had no effect on the fecal microbiome and endotoxin concentration. Nevertheless, early-fed chickens were heavier than the non-early-fed chickens until slaughter age. All these factors need to be further investigated under commercial conditions, and the genetic strain seems to be the most influential factor to be considered.

## Data Availability

The datasets presented in this study can be found in online repositories. The names of the repository/repositories and accession number(s) can be found in the article/Supplementary Material.
