# Supplementary figures and images for: Host genotype affects endotoxin release in excreta of broilers at slaughter age
Source: Front Genet. 2023 Jun 8;14:1202135. doi: 10.3389/fgene.2023.1202135 (PMC10285083; doi:10.3389/fgene.2023.1202135)

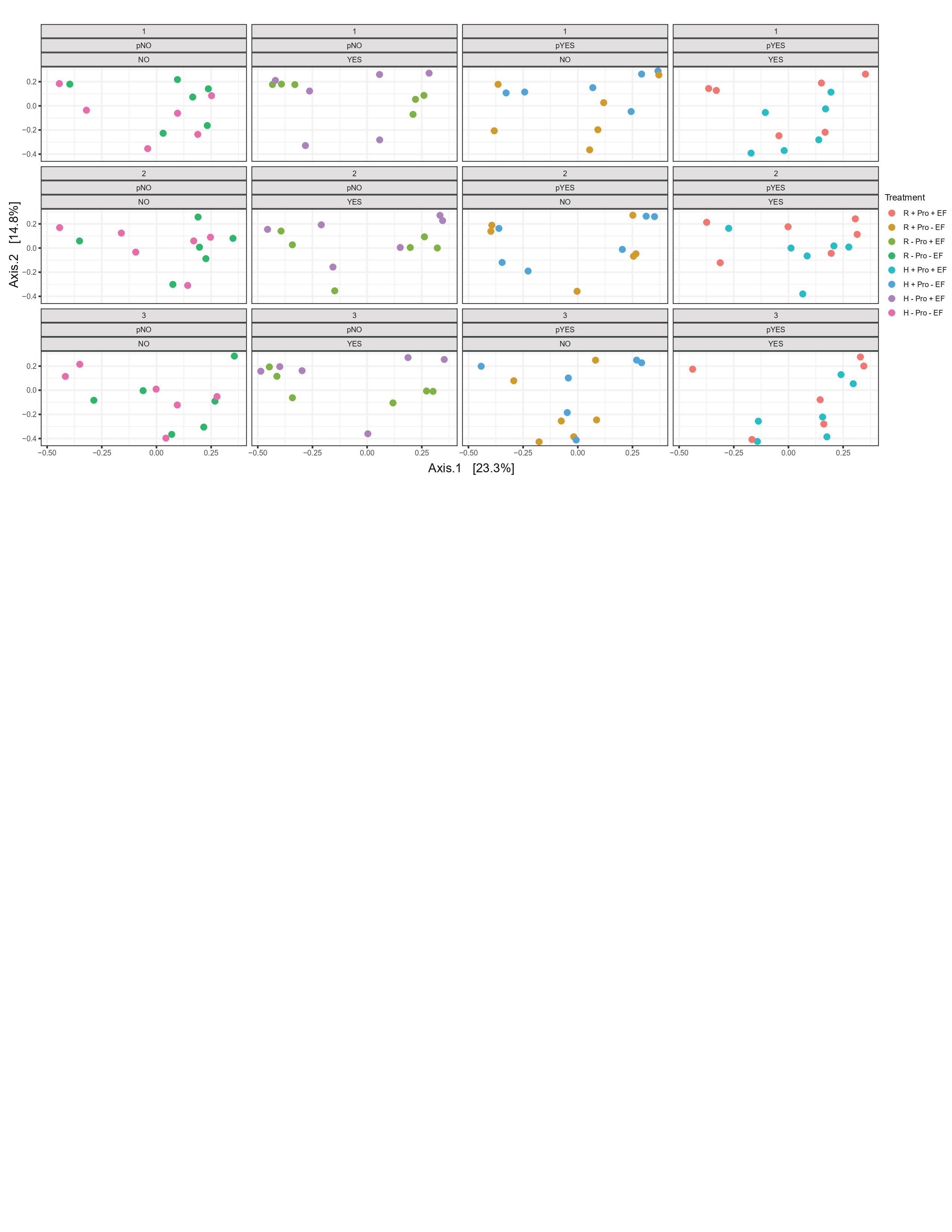

Supplement: Supplementary file 1 [file Image3.JPEG]

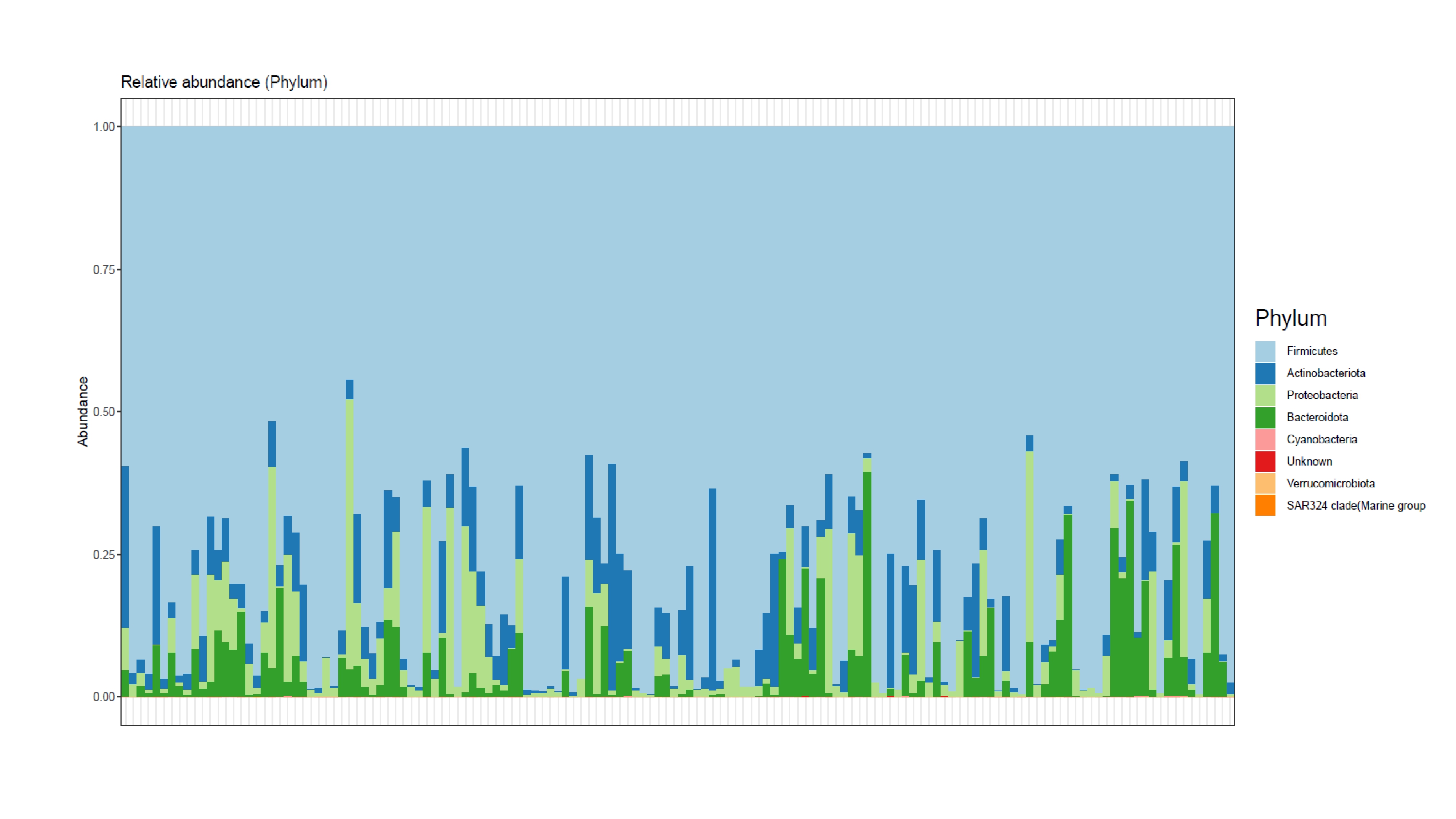

Supplement: Supplementary file 3 [file Image1.JPEG]

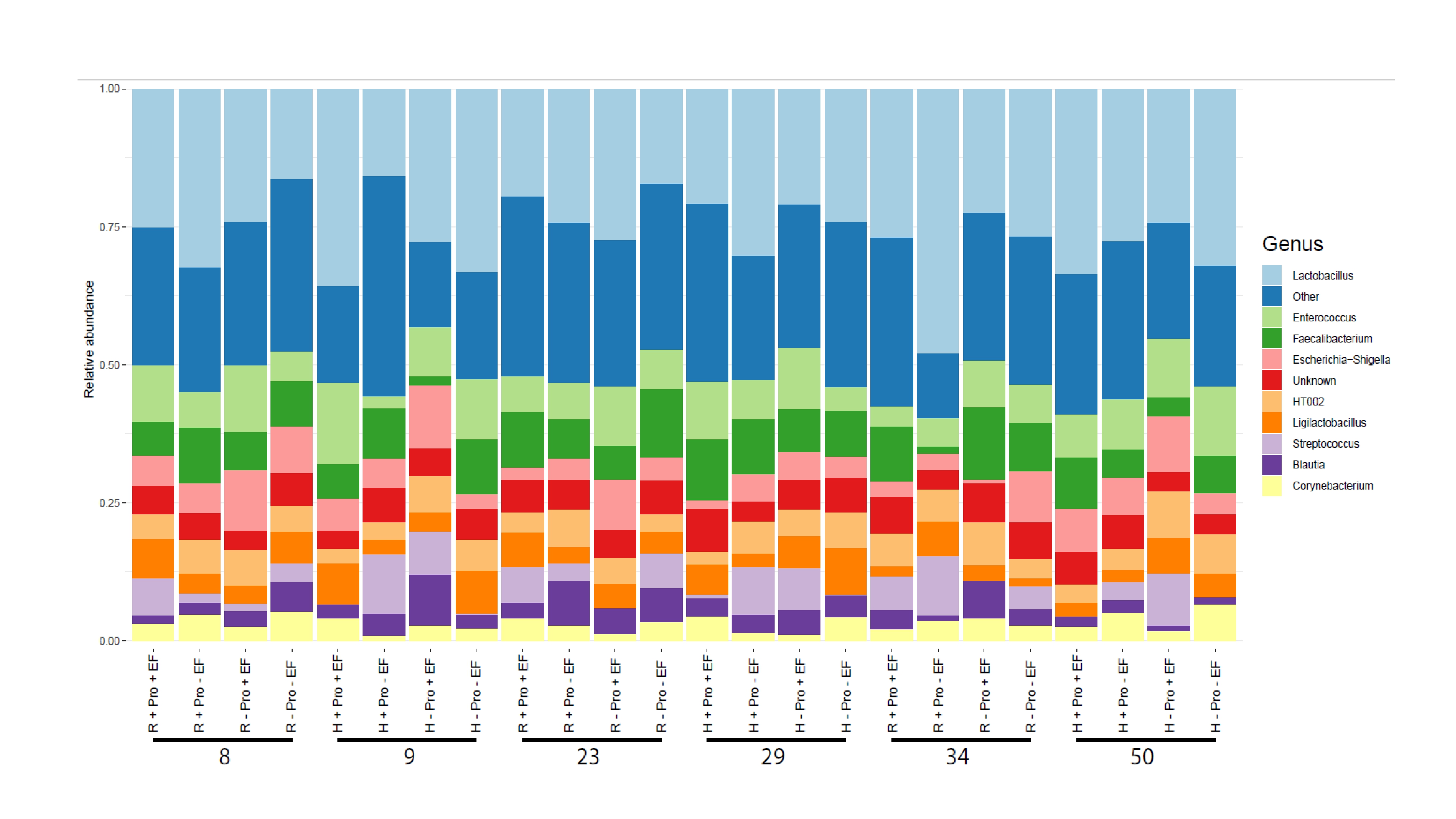

Supplement: Supplementary file 4 [file Image2.JPEG]
