# Supplementary material for: Host genotype affects endotoxin release in excreta of broilers at slaughter age
Source: Front Genet. 2023 Jun 8;14:1202135. doi: 10.3389/fgene.2023.1202135 (PMC10285083; doi:10.3389/fgene.2023.1202135)
Supplement: Supplementary file 2 [file Table1.DOCX]

**Table S1.** Composition and calculated components of the experimental diets fed to broiler chickens.

| **Ingredients (g/kg)** | **Prestarter** | **Starter (0-14 d)** | **Grower (14-37 d)** | **Finisher (37-51 d)** |
| --- | --- | --- | --- | --- |
| Corn | 303.70 | 279.45 | 250.75 | 164.40 |
| Soybean meal 480 cp | 223.00 | 300.00 | 210.00 | 175.00 |
| Wheat + xyl (105 ME) | 300.00 | 300.00 | 400.00 | 500.00 |
| Corn gluten meal | 20.00 | - | - | - |
| Rapeseed meal 330 CP | - | 20.00 | 30.00 | 40.00 |
| Sunflower meal Hipro | - | 20.00 | 30.00 | 40.00 |
| Oats | - | 20.00 | 20.00 | 20.00 |
| Potato protein (Ash < 10) | 30.00 | - | - | - |
| Wheat middlings | 50.00 | - | - | - |
| Soybean oil | 38.50 | 30.00 | 35.20 | 43.50 |
| Premix (mais) | 5.00 | 5.00 | 5.00 | 5.00 |
| Lime fine | 11.00 | 10.80 | 8.60 | 5.60 |
| Monocalciumphosphate | 9.30 | 6.70 | 3.50 | 0.00 |
| Salt | 1.90 | 1.70 | 1.30 | 1.30 |
| NaHCO_3_ | 2.90 | 3.10 | 3.00 | 3.00 |
| L-Lysine HCl | 2.20 | 1.50 | 1.35 | 1.40 |
| DL-methionine | 1.65 | 1.70 | 1.20 | 0.75 |
| Betaine HCl | 1.00 | - | - | - |
| L-threonine | - | 0.00 | 0.05 | 0.00 |
| L-valine | 0.00 | 0.00 | 0.00 | 0.00 |
| Axtra PHY 1000 ftu | 0.05 | 0.05 | 0.05 | 0.05 |
| **Calculated contents (g/kg)** |  |  |  |  |
| Dry matter | 877.00 | 875.50 | 874.20 | 873.6 |
| Ash | 49.80 | 51.10 | 41.90 | 35.00 |
| Crude protein | 211.90 | 218.20 | 190.30 | 184.50 |
| Crude fat | 65.00 | 55.50 | 60.00 | 66.20 |
| Crude fiber | 24.80 | 30.70 | 32.20 | 34.80 |
| Carbohydrates | 524.40 | 518.80 | 549.10 | 553.00 |
| Starch | 395.80 | 376.00 | 418.20 | 422.60 |
| Sugars | 39.90 | 47.30 | 41.60 | 40.50 |
| NDF | 97.40 | 99.60 | 104.90 | 109.00 |
| ADF | 35.70 | 43.90 | 46.30 | 49.80 |
| Calcium | 8.50 | 8.30 | 6.80 | 5.10 |
| Phosphorous, total | 5.90 | 5.60 | 4.70 | 3.90 |
| Phosphorous, available | 5.30 | 4.80 | 4.00 | 3.20 |
| Phosphorous, dig pigs | 2.90 | 2.50 | 1.80 | 1.20 |
| Phosphorous, dig poultry | 4.80 | 4.40 | 3.70 | 3.0 |
| Calcium/dP poultry | 1.77 | 1.89 | 1.84 | 1.68 |
| Magnesium | 1.50 | 1.70 | 1.50 | 1.50 |
| Potassium | 7.80 | 9.30 | 7.90 | 7.50 |
| Sodium | 1.60 | 1.60 | 1.40 | 1.40 |
| Chloride | 2.20 | 1.70 | 1.50 | 1.50 |
| Base-excess (meq/kg) | 205.80 | 259.30 | 222.20 | 211.50 |
| Linolic acid | 29.70 | 24.80 | 27.00 | 30.00 |
| Isoleucine | 8.96 | 9.15 | 7.74 | 7.39 |
| Leucine | 18.28 | 17.08 | 14.67 | 13.74 |
| Lysine | 12.37 | 12.38 | 10.14 | 9.56 |
| Methionine | 5.13 | 5.02 | 4.20 | 3.70 |
| Cystine | 3.61 | 3.74 | 3.43 | 3.42 |
| Meth.+Cyst. | 8.74 | 8.76 | 7.63 | 7.12 |
| Phenylalanine | 10.72 | 10.63 | 9.12 | 8.73 |
| Tyrosine | 7.98 | 7.55 | 6.37 | 6.00 |
| Phenyl. + Tyr. | 18.69 | 18.17 | 15.48 | 14.73 |
| Threonine | 8.04 | 8.02 | 6.90 | 6.55 |
| Tryptophan | 2.47 | 2.63 | 2.27 | 2.22 |
| Valine | 10.24 | 10.22 | 8.86 | 8.55 |
| Arginine | 12.78 | 14.40 | 12.11 | 11.56 |
| Histidine | 5.37 | 5.74 | 4.94 | 4.72 |
| Alanine | 10.36 | 9.96 | 8.61 | 8.07 |
| Aspartic acid | 20.15 | 21.30 | 17.30 | 16.06 |
| Glutamic acid | 39.93 | 42.50 | 38.68 | 38.81 |
| Glycine | 8.78 | 9.26 | 8.13 | 7.96 |
| Proline | 13.67 | 13.41 | 12.44 | 12.41 |
| Serine | 10.51 | 10.80 | 9.26 | 8.87 |
| Glycine + Serine | 19.28 | 20.06 | 17.39 | 16.83 |
