# Supplementary material for: Host genotype affects endotoxin release in excreta of broilers at slaughter age
Source: Front Genet. 2023 Jun 8;14:1202135. doi: 10.3389/fgene.2023.1202135 (PMC10285083; doi:10.3389/fgene.2023.1202135)
Supplement: Supplementary file 5 [file Table2.DOCX]

**Table S2.** List of the bacterial groups analyzed in the current study.

| **Genus** | **Gram** |
| --- | --- |
| *[Clostridium] innocuum* group | Gram Positive |
| *[Eubacterium] coprostanoligenes* group | Gram Positive |
| *[Eubacterium] hallii* group | Gram Positive |
| *[Ruminococcus] gauvreauii* group | Gram Positive |
| *[Ruminococcus] torques* group | Gram Positive |
| *Abiotrophia* | Gram Positive |
| *Achromobacter* | Gram Negative |
| *Acinetobacter* | Gram Negative |
| *Actinobacillus* | Gram Negative |
| *Actinomyces* | Gram Positive |
| *Aerococcus* | Gram Positive |
| *Aeromonas* | Gram Negative |
| *Aggregatibacter* | Gram Negative |
| *Alcaligenes* | Gram Negative |
| *Alistipes* | Gram Negative |
| *Anaerococcus* | Gram Positive |
| *Anaerostipes* | Gram Positive |
| *Anaerotruncus* | Gram Positive |
| *Arthrobacter* | Gram Positive |
| *Atopobium* | Gram Positive |
| *Bacillus* | Gram Positive |
| *Bacteroides* | Gram Negative |
| *Barnesiella* | Gram Negative |
| *Bifidobacterium* | Gram Positive |
| *Blautia* | Gram Positive |
| *Bordetella* | Gram Negative |
| *Brachybacterium* | Gram Positive |
| *Brevibacterium* | Gram Positive |
| *Brevundimonas* | Gram Negative |
| *Burkholderia* | Gram Negative |
| *Butyricicoccus* | Gram Positive |
| *CAG-56* | Gram Positive |
| *Campylobacter* | Gram Negative |
| *Capnocytophaga* | Gram Negative |
| *Cedecea* | Gram Negative |
| *CHKCI002* | ND |
| *Chlamydiae* | Gram Negative |
| *Christensenellaceae R-7 group* | Gram Variable |
| *Citrobacter* | Gram Negative |
| *Clostridia* | Gram Positive |
| *Clostridia UCG-014* | Gram Positive |
| *Clostridia vadinBB60 group* | Gram Positive |
| *Clostridium sensu stricto 1* | Gram Positive |
| *Colidextribacter* | Gram Negative |
| *Collinsella* | Gram Positive |
| *Corynebacterium* | Gram Positive |
| *Cronobacter* | Gram Negative |
| *Cupriavidus* | Gram Negative |
| *Defluviitaleaceae UCG-011* | Gram Positive |
| *Delftia* | Gram Negative |
| *Dermabacter* | Gram Positive |
| *Dermatophilus* | Gram Positive |
| *Dietzia* | Gram Positive |
| *Dorea* | Gram Positive |
| *Edwardsiella* | Gram Negative |
| *Eikenella* | Gram Negative |
| *Eisenbergiella* | Gram Variable |
| *Elizabethkingia* | Gram Negative |
| *Enterobacter* | Gram Negative |
| *Enterococcaceae* | Gram Positive |
| *Enterococcus* | Gram Positive |
| *Erysipelatoclostridium* | Gram Positive |
| *Erysipelotrichaceae* | Gram Positive |
| *Escherichia-Shigella* | Gram Negative |
| *Eubacterium* | Gram Positive |
| *Faecalibacterium* | Gram Negative |
| *Family XIII AD3011 group* | Gram Positive |
| *Flavonifractor* | Gram Variable |
| *Fournierella* | Gram Negative |
| *Fusobacterium* | Gram Negative |
| *Gardnerella* | Gram Positive |
| *Gardnerella* | Gram Negative |
| *GCA-900066575* | Gram Positive |
| *Gemella* | Gram Positive |
| *Gordonia* | Gram Positive |
| *Gordonibacter* | Gram Positive |
| *Granulicatella* | Gram Positive |
| *Haemophilus* | Gram Negative |
| *Hafnia* | Gram Negative |
| *Helicobacter* | Gram Negative |
| *HT002* | Gram Positive |
| *Hydrogenoanaerobacterium* | Gram Negative |
| *Incertae Sedis* | Gram Positive |
| *Intestinimonas* | Gram Positive |
| *Kingella* | Gram Negative |
| *Klebs.* | Gram Negative |
| *Klebsiella* | Gram Negative |
| *Kluyvera* | Gram Negative |
| *Lachnoclostridium* | Gram Positive |
| *Lachnospiraceae* | Gram Positive |
| *Lachnospiraceae FE2018 group* | Gram Positive |
| *Lachnospiraceae NK4A136 group* | Gram Positive |
| *Lachnospiraceae UCG-004* | Gram Positive |
| *Lachnospiraceae UCG-008* | Gram Positive |
| *Lactobacillaceae* | Gram Positive |
| *Lactobacillales* | Gram Positive |
| *Lactobacillus* | Gram Positive |
| *Lactococcus* | Gram Positive |
| *Legionella* | Gram Negative |
| *Leifsonia* | Gram Positive |
| *Leuconostoc* | Gram Positive |
| *Ligilactobacillus* | Gram Positive |
| *Limosilactobacillus* | Gram Positive |
| *Listeria* | Gram Positive |
| *Marvinbryantia* | Gram Positive |
| *Megamonas* | Gram Negative |
| *Merdibacter* | Gram Negative |
| *Micrococcus* | Gram Positive |
| *Mobiluncus* | Gram Positive |
| *Mobiluncus* | Gram Negative |
| *Monoglobus* | Gram Positive |
| *Moraxella* | Gram Negative |
| *Morganella* | Gram Negative |
| *Myroides* | Gram Negative |
| *Negativibacillus* | Gram Negative |
| *Neisseria* | Gram Negative |
| *NK4A214 group* | Gram Positive |
| *Nocardia* | Gram Positive |
| *Oligella* | Gram Negative |
| *Oscillibacter* | Gram Negative |
| *Oscillospiraceae* | Gram Negative |
| *Paenibacillus* | Gram Positive |
| *Pantoea* | Gram Negative |
| *Parabacteroides* | Gram Negative |
| *Parascardovia* | Gram Positive |
| *Pasteurella* | Gram Negative |
| *Pediococcus* | Gram Positive |
| *Peptococcus* | Gram Positive |
| *Peptoniphilus* | Gram Positive |
| *Peptostreptococcaceae* | Gram Positive |
| *Peptostreptococcus* | Gram Positive |
| *Phascolarctobacterium* | Gram Negative |
| *Plesiomonas* | Gram Negative |
| *Pleuralibacter* | Gram Negative |
| *Prevotella* | Gram Negative |
| *Propionibacterium* | Gram Positive |
| *Propioniferax* | Gram Positive |
| *Proteus* | Gram Negative |
| *Providencia* | Gram Negative |
| *Pseudomonas* | Gram Negative |
| *Pseudoramibacter* | Gram Positive |
| *Psychrobacter* | Gram Negative |
| *Pygmaiobacter* | Gram Positive |
| *Ralstonia* | Gram Negative |
| *Raoultella* | Gram Negative |
| *RF39* | x |
| *Rhodococcus* | Gram Positive |
| *Romboutsia* | Gram Positive |
| *Rothia* | Gram Positive |
| *Ruminococcaceae* | Gram Positive |
| *Ruminococcus* | Gram Positive |
| *Salmonella* | Gram Negative |
| *Scardovia* | Gram Positive |
| *Sellimonas* | Gram Positive |
| *Serratia* | Gram Negative |
| *Shewanella* | Gram Negative |
| *Shigella* | Gram Negative |
| *Shuttleworthia* | Gram Positive |
| *Siccibacter* | Gram Negative |
| *Sphingomonas* | Gram Negative |
| *Staphylococcus* | Gram Positive |
| *Stenotrophomonas* | Gram Negative |
| *Streptococcus* | Gram Positive |
| *Subdoligranulum* | Gram Variable |
| *Suttonella* | Gram Negative |
| *Tatumella* | Gram Negative |
| *Trueperella* | Gram Positive |
| *Turicibacter* | Gram Positive |
| *Tyzzerella* | Gram Negative |
| *UC5-1-2E3* | Gram Positive |
| *UCG-005* | Gram Positive |
| *Veillonella* | Gram Negative |
| *Vibrio* | Gram Negative |
| *Weeksella* | Gram Negative |
| *Weissella* | Gram Positive |
| *Yersinia* | Gram Negative |
