# Supplementary material for: Host genotype affects endotoxin release in excreta of broilers at slaughter age
Source: Front Genet. 2023 Jun 8;14:1202135. doi: 10.3389/fgene.2023.1202135 (PMC10285083; doi:10.3389/fgene.2023.1202135)
Supplement: Supplementary file 6 [file Table3.DOCX]

**Table S3.** Effects of time point on endotoxin concentration (EU/ml) in pooled cloacal swabs (N=144 in total) collected from broiler chickens.

| Time points | Endotoxins (EU/ml per broiler) | SEM^1^ | P-value |
| --- | --- | --- | --- |
| Time point 1 (target BW^2^ = 200 g) | 477.1 | 100.6 | < 0.01 |
| Time point 2 (target BW = 1 kg) | 580.3 |  |  |
| Time point 3 (target BW = 2.5 kg) | 1063.7 |  |  |

^1^SEM = standard error of the mean; ^2^BW= body weight.
