# Supplementary material for: Host genotype affects endotoxin release in excreta of broilers at slaughter age
Source: Front Genet. 2023 Jun 8;14:1202135. doi: 10.3389/fgene.2023.1202135 (PMC10285083; doi:10.3389/fgene.2023.1202135)
Supplement: Supplementary file 7 [file Table4.DOCX]

**Table S4.** Effects of broiler breeder strain (fast-growing Ross 308 and slower-growing Hubbard JA757), addition of pre/probiotic to the diet and early feeding on dry matter (DM) of the litter measured at different feeding phases and time points (LSmeans± SEM^1^).

| **Parameter** | **Strain** | | **SEM** | ***P*-value** | **Probiotic** | | **SEM** | ***P*-value** | **Early feeding** | | **SEM** | ***P*-value** |
| --- | --- | --- | --- | --- | --- | --- | --- | --- | --- | --- | --- | --- |
|  | **Ross 308** | **Hubbard JA757** |  |  | **Yes** | **No** |  |  | **Yes** | **No** |  |  |
| **DM of litter (%)** |  |  |  |  |  |  |  |  |  |  |  |  |
| Time point 1 **(target BW^2^ = 200 g)** | 62.22 | 66.62 | 1.57 | 0.81 | 63.0 | 65.83 | 1.57 | 0.87 | 62.34 | 66.50 | 1.57 | 0.82 |
| Time point 2 **( target BW = 1 kg)** | 62.49 | 62.62 | 1.31 | 0.99 | 62.68 | 62.43 | 1.31 | 0.99 | 62.08 | 63.03 | 1.31 | 0.96 |
| Time point 3 **( target BW = 2.5 kg)** | 46.42 | 42.92 | 0.96 | 0.85 | 43.67 | 45.67 | 0.96 | 0.91 | 44.06 | 45.28 | 0.96 | 0.94 |

^1^SEM= standard error of the mean; ^2^BW = body weight.
